# Balancing Harvesting and Conservation: Demographic Responses of a Threatened Palm to Anthropogenic Disturbance

**DOI:** 10.1002/ece3.72882

**Published:** 2026-01-07

**Authors:** Eduardo T. B. Mendes, Rita C. Q. Portela

**Affiliations:** ^1^ Programa de Pós‐Graduação em Ecologia, Departamento de Ecologia, Instituto de Biologia Universidade Federal do Rio de Janeiro Rio de Janeiro Brazil; ^2^ Departamento de Ecologia, Instituto de Biologia Universidade Federal do Rio de Janeiro Rio de Janeiro Brazil

**Keywords:** demography, *Euterpe edulis*, integral projection models, population dynamics, resource harvesting

## Abstract

The expansion of human activities into natural ecosystems has driven demographic changes across numerous species. In terrestrial environments, land‐use change and the harvesting of forest resources are major drivers of population decline and extinction, particularly among species dependent on continuous forest cover. Despite advances in demographic research, the long‐term population responses of many species to these pressures remain poorly understood, especially in landscapes subject to intense deforestation. This study evaluated the dynamics of palm populations not directly harvested but exposed to anthropogenic disturbances, using numerical simulations to assess the demographic effects of varying intensities and frequencies of palm heart and fruit harvesting. Over a 19‐year period, Integral Projection Models revealed declining population growth rates, with the survival of large individuals exerting the greatest influence on population trajectories. Simulations indicated that harvesting of palm heart substantially exacerbated population decline, particularly when targeting immature and mature individuals at high intensity and frequency. In contrast, harvesting of fruits, which does not result in direct mortality, had a comparatively lower demographic impact and may be sustainable under specific conditions. These findings highlight the importance of regulating resource extraction and implementing conservation measures, especially in fragmented landscapes, to ensure the persistence of species vulnerable to anthropogenic disturbance. The modeling framework applied here provides a valuable approach for evaluating demographic responses to human activities in other plant species and ecosystems. Furthermore, this type of study is crucial for informing sustainable management policies and for offering valuable insights into balancing resource extraction with conservation needs in fragmented landscapes.

## Graphical abstract

Nineteen years of demographic data reveal a consistent decline of *Euterpe edulis* in forest fragments, highlighting the species' vulnerability. While the population shows resilience to fruit harvesting, palm heart extraction leads to a demographic collapse. Ensuring the survival of large individuals and controlling illegal palm heart harvesting are critical for maintaining population stability in forest remnants.

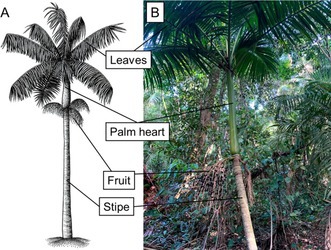

## 1 Introduction

Several ecosystems are currently experiencing pressures from anthropogenic activities (Lindenmayer et al. 2017). In terrestrial environments, one of these pressures primarily arises from the overharvesting of species or their parts for human consumption. This practice drives population declines (McRae et al. 2022), with many species now facing high extinction risks (IUCN 2023). Therefore, studies assessing how populations of species under illegal harvesting pressure change over time are both urgent and essential for the development of effective conservation and management strategies.

It is estimated that 50,000 species are currently harvested for human consumption, many of which are sourced from natural environments (IPBES 2022). Because of the lack of regulation of such practices, several species' populations are harvested unsustainably, leading to their decline or even extinction (Ticktin 2004; Fernandez et al. 2012). Harvesting directly affects populations by influencing vital rates and resulting in demographic changes (Schmidt et al. 2011). In particular, the removal of whole individuals or vital parts directly reduces survival and, when applied to reproductive individuals, also decreases fecundity. Similarly, the harvest of reproductive structures (e.g., seeds, flowers, and gonads) compromises population replenishment by reducing fecundity (Ticktin 2004; Schmidt et al. 2011). The impact of harvesting on populations depends on each species' life history. In long‐lived species, changes in the survival rate of larger individuals have drastic demographic consequences due to the greater importance of this vital rate. In contrast, for short‐lived species, changes in fecundity rates have a greater impact, given their greater demographic significance (Franco and Silvertown 2004; Bucharova et al. 2025). On the other hand, resource harvesting has historically been a means of subsistence and income generation for human populations (Wells et al. 2023). Therefore, conservation strategies that ensure both the persistence of harvested species and the subsistence of human communities are essential to implement.

Worldwide, harvesting of natural resources has been practiced for centuries (Salo et al. 2013). In economically poorer regions of the globe, species populations are more heavily harvested and consequently face a greater risk of extinction (Price and Gittleman 2007; Weinbaum et al. 2013; McRae et al. 2022). This increased risk in these regions arises from the lack of laws and management strategies for biodiversity control (Marsh et al. 2022), along with the greater need for resource extraction to support subsistence and supplement financial income (Morton et al. 2021). To develop viable conservation strategies for biodiversity, it is essential to understand not only the target species but also the social context in which the harvested populations occur (Charnley et al. 2023).

The Atlantic Forest is a prime example of this situation, as it is highly impacted by anthropogenic activities, such as illegal harvesting of forest resources (Marques and Grelle 2021). The biome extends along Brazil's eastern coastal zone, where the concentration of urban areas facilitates the activities of illegal harvesters (Galetti and Fernandez 1998; Sánchez‐Mercado et al. 2016). Today, only about 23% of the biome's original vegetation remains, mostly as forest fragments, and only 30% of the biome is under protection, with 9% designated as strictly protected areas, where human activities are prohibited, and 21% designated for sustainable use, where human activities are allowed but subject to strict regulations (Ribeiro et al. 2009; Rezende et al. 2018; Vancine et al. 2024). As a result, many species have experienced severe population declines, facing critical risks of local extinction (Martinelli and Moraes 2013). In 2006, the Atlantic Forest Law (Law No. 11,428/2006) was enacted, establishing guidelines and regulations to combat illegal harvesting and to enable the conservation, protection, and regeneration of the Atlantic Forest's natural resources.

*Euterpe edulis* Mart. (Arecaceae) is a long‐lived species native to the Atlantic Forest and also occurs in the ecotone between the Atlantic Forest and the Brazilian Savanna, dependent on preserved forest habitats for survival (Henderson et al. 1995). The species is highly important and considered a keystone in the ecosystem, providing food resources for bird and mammal seed dispersers, especially during periods of resource scarcity. Due to pressures from deforestation and resource harvesting, the species' populations have declined in the wild (Souza and Prevedello 2020; Leal et al. 2021), and it is currently considered vulnerable to extinction (Martinelli and Moraes 2013). The largest populations of the species are found within protected areas and in larger, continuous forest patches, whereas populations in unprotected small forest fragments are significantly reduced (Melito et al. 2014; Souza and Prevedello 2020). The species is harvested for its palm heart (i.e., apical meristem) and fruits for human consumption. Because *Euterpe edulis* is a threatened species, the harvesting of palm heart is currently prohibited in Brazil (Law No. 11,428/2006), as it involves killing individuals, making it a demographically unsustainable form of harvesting. In contrast, fruit harvesting allows individuals to survive, making it demographically sustainable (Ticktin 2004; Mendes et al. 2022).

Several studies have investigated how populations of *Euterpe edulis* respond to anthropogenic pressures, both within and outside protected areas (Souza and Prevedello 2020; Melito et al. 2014). Regarding exploitation, studies have assessed its impacts on the community, including seed rain (Muler et al. 2014) and species interactions (Pizo and Vieira 2004). Reis et al. (2000) also evaluated the potential for sustainable harvesting of *E. edulis* based on the structure of large populations in protected areas, which has informed regulations on the species' extraction. However, information remains scarce on small populations, commonly found in forest fragments, the most representative condition of the Atlantic Forest, and how they respond to fruit and palm heart harvesting under different scenarios. Advancing this understanding, together with existing knowledge, could improve conservation strategies and guide the management of resources derived from the species, thereby supporting its persistence in the biome.

Based on the information above, this study aimed to evaluate the population dynamics of three *Euterpe edulis* populations subjected to anthropogenic pressures, by simulating different harvesting scenarios for palm heart and fruits. To achieve this, the following questions were proposed: (a) how are the populations performing demographically in the long term in the absence of harvesting? (b) how do the populations respond demographically when simulating palm heart harvesting occurring only in mature individuals, across different removal intensities and frequencies of harvesting events over time? (c) how do the populations respond demographically when simulating palm heart harvesting occurring in all individuals ≥ 65 mm (reproducing or not)? and (d) how do the populations respond demographically to fruit harvesting? The following hypotheses are considered: (a) as a long‐lived species, populations will experience a more pronounced demographic impact under the scenario of removing the mature and immature individuals (≥ 65 mm), since the survival of these individuals has the greatest demographic importance; (b) in contrast, fruit harvesting is expected to have a lower demographic impact compared to palm heart exploitation, regardless of the intensity and frequency of harvesting events, due to the limited demographic influence of this vital rate.

## 2 Material and Methods

### 2.1 Study Sites

**FIGURE 1:**
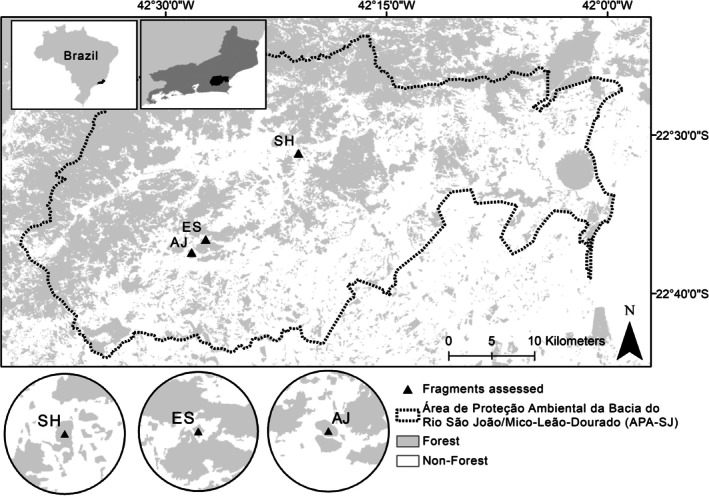
The three fragments assessed: Santa Helena (SH), Afetiva‐Jorge (AJ) and Estreito (ES), in Área de Proteção Ambiental da Bacia do Rio São João/Mico‐Leão‐Dourado (APA‐SJ), Rio de Janeiro state, Brazil. Buffers with a radius of 2000 m from the centroid show the surroundings of each fragment in detail.

The study was conducted in three small lowland Atlantic Forest fragments located in the state of Rio de Janeiro, southeastern Brazil, where each forest fragment comprised a population of the species *Euterpe edulis*. The forest fragments were identified as Santa Helena (SH), Estreito (ES; 21 ha), and Afetiva‐Jorge (AJ; 19 ha) (Figure 1). All three forest fragments are on private properties within a sustainable use protected area (APA‐SJ, IUCN category V), where human activities (e.g., residences, agriculture, etc.) are allowed but controlled to prevent environmental damage. The three areas support a high diversity of plant species, with a dominance of Fabaceae, Euphorbiaceae, Rubiaceae, Lauraceae, Moraceae, Myrtaceae, Annonaceae, and Sapotaceae taxa (Carvalho et al. 2008). The soil is classified as Red‐Yellow Latosol, characteristic of low‐altitude, non‐flooded forests, deep and well‐drained, but susceptible to erosion (Primo and Völker 2003). The climate is classified as Walter and Lieth's equatorial type (Walter 1971), with an annual precipitation oscillating around 1500 and 2000 mm (Primo and Völker 2003).

The SH population is located within a 57‐ha forest fragment, situated on private property and bordered by a high‐traffic highway (BR‐101). The fragment contains walking trails, but no evidence of wildlife presence was observed. During the sampling period, no signs of human harvesting of *E. edulis* palm heart or fruits were recorded. The terrain is predominantly flat, with occasional slopes.

The ES population occurs in a 21‐ha forest fragment on private property, with no nearby roads or urban centers. The fragment is entirely fenced, with no internal trails and no signs of animal intrusion. Throughout the sampling period, there was no evidence of human harvesting of *E. edulis* palm heart or fruits. The terrain is mostly flat, with occasional slopes.

The AJ population is found in a 19‐ha forest fragment located on private property, isolated from roads and urban areas. Walking trails are present within the fragment, and evidence of cattle presence was detected. During the sampling period, occasional instances of illegal harvesting of *E. edulis* palm hearts were observed, resulting in a total of 20 mature and immature individuals harvested over the 19 years of the study. The terrain is mostly flat, with occasional slopes.

### 2.2 Focus Species

**FIGURE 2:**
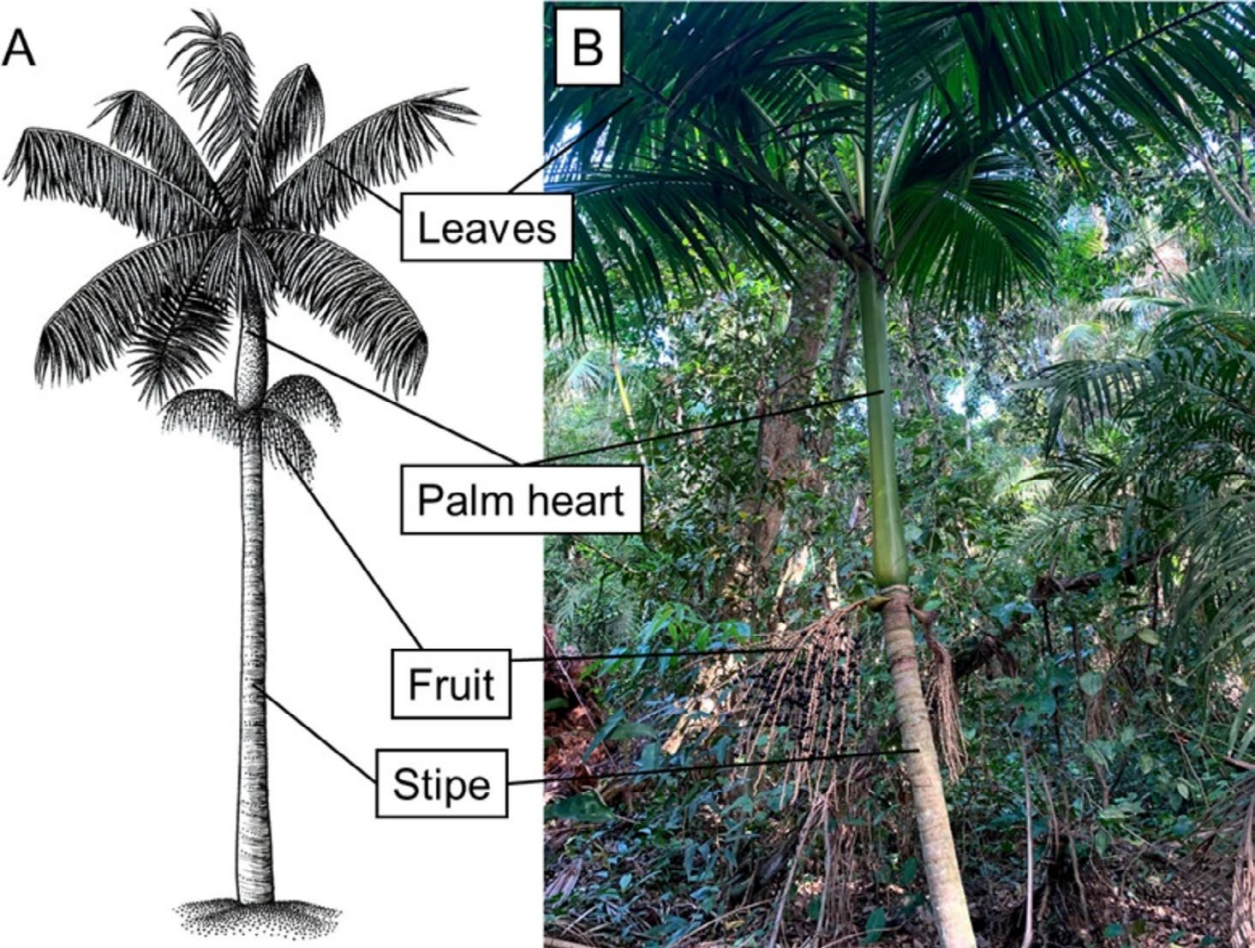
Representation of the aerial structures of a mature individual of the palm species *Euterpe edulis*, with a schematic drawing (A) and a field specimen (B). *Source:* (A)—drawing by Luis Gustavo Barretto Rodrigues; (B)—photograph by Guilherme Gama.

The focal species of this study was *Euterpe edulis*, a monoecious, single‐stemmed, shade‐tolerant palm, with a wide distribution along the Brazilian Atlantic Forest (Henderson et al. 1995; Figure 2). With slow growth, individuals of the species typically begin reproducing around 23 years of age and have a lifespan of approximately 38 years (unpublished results). Each mature individual can produce around 1500 fruits per year, which are consumed by 58 species of birds and 21 species of mammals (Matos and Watkinson 1998; Galetti et al. 2013). Due to intense human harvesting pressure on the resources provided by the species (e.g., fruits and palm heart), *E. edulis* populations have been drastically reduced in the wild, and the species is currently considered vulnerable to extinction (Martinelli and Moraes 2013).

### 2.3 Data Collection

Demographic data for the three *E. edulis* populations were sampled annually between 2005 and 2024, except in 2008 and 2009, when no sampling was conducted. All annual censuses were conducted in September, which corresponds to the period immediately after fruiting and dispersal. In each of the three populations, nine 900 m^2^ plots were permanently established and systematically distributed across three blocks, covering a total area of 0.81 ha per population. Each block contained three sampling plots, spaced 50 m apart, and separated by a distance of 100 m from the next block and at least 50 m from the fragment edge. All individuals of *E. edulis* were marked and numbered with aluminum tags. Starting in 2006, those individuals that had recently germinated and had not yet received a numbered tag were also identified (i.e., recruits). Annually, the diameter at ground level (DGL) of each individual was measured using a Mitutoyo digital caliper, and the presence or absence of reproductive structures (i.e., flower or fruit bunches) was recorded. The presence of reproductive structures indicated that the individuals were mature.

A total of 2059 *E. edulis* individuals were recorded in the three forest fragments over 19 years of monitoring. The mean density of *E. edulis* (ind/ha) per year in each fragment was 117.0 ind/ha (±30.0) for SH, followed by 162.1 ind/ha (±132.1) for ES, and 93.0 ind/ha (±17.0) for AJ. The mean density of mature individuals was 14.8 ind/ha (±5.4) in SH, 7.7 ind/ha (±1.7) in ES, and 17.2 ind/ha (±5.7) in AJ. The mean density of recruits in SH was 16.0 ind/ha (±31.5), 67.2 ind/ha (±67.0) in ES, and 22.3 ind/ha (±17.5) in AJ. The DGL size ranged from 1.35 mm to 148.27 mm in SH, 1.71 mm to 148.23 mm in ES, and 1.19 mm to 133.52 mm in AJ.

### 2.4 Harvesting Simulations

Four scenarios were simulated to assess the demographic response of *E. edulis* to harvesting. These scenarios were developed based on previous information (e.g., Matos and Watkinson 1998; Freckleton et al. 2003; Mendes et al. 2022), considering size as the minimum diameter from which individuals of the species exhibit a palm heart with relevant harvesting potential.

Scenario 1 (control) involved no harvesting, but populations were subjected to past effects resulting from deforestation and habitat fragmentation; Scenario 2 involved the harvesting of only mature individuals; Scenario 3 involved the harvesting of both mature and immature individuals with a DGL ≥ 65 mm; and Scenario 4 involved the harvesting of fruits. For Scenario 3, based on our personal observations, it was determined that individuals with a diameter of 65 mm already have a palm heart suitable for consumption.

For Scenarios 2, 3, and 4, harvesting was evaluated at three removal intensities from the target classes: 30%, 60%, and 100%. Different harvesting event frequencies were assessed over a 10‐year period. For low frequency of events, harvesting was considered to occur only once in 10 years; for medium frequency, it occurred three times in 10 years; and for high frequency, it occurred five times in 10 years. The 10‐year simulation horizon was chosen because it represents a reasonable interval for detecting population trends in species with intermediate to long lifespans, such as *E. edulis* , and because it is a temporal unit frequently used as a criterion in population trend assessments (Mace et al. 2008).

### 2.5 Data Analysis

#### 2.5.1 Stochastic Dynamics

The population dynamics of *E. edulis* under harvesting scenarios were evaluated using stochastic demographic analysis based on Integral Projection Models (IPM; Easterling et al. 2000). Regardless of the harvesting scenario, the stochastic analysis requires, as a first step, the estimation of deterministic kernels for each transition year in each population.

For Scenario 1 (control), each kernel (hereafter referred to as control kernel) was estimated by considering the variation in population structure during the sampling interval, according to the equation:

$$n\;\left(y,t+1\right)=\int_{L}^{U}K\left(y,x\right)n\left(x,t\right)dx$$

where *n* (*y*, *t* + 1) represents the population structure at time *t* + 1, and *n* (*x*, *t*) represents the population structure at time *t*, based on survival, growth, regression, and recruitment contributions over the sampling interval. *K* is the deterministic kernel *k* (*y*, *x*) that includes all possible transitions in individual size from time *t*, represented by *x*, to time *t* + 1, represented by *y*, considering the spectrum of all possible individual sizes, with *L* as the minimum size and *U* as the maximum size. To estimate the kernel, two sub‐kernels, *P* (*x*, *y*) and *F* (*x*, *y*), were computed. *P* (*x*, *y*) includes all survival and growth transitions of individuals, while *F* (*x*, *y*) includes all fecundity probabilities (Ellner et al. 2016). The survival transition was estimated by determining whether each individual at time *t* survived until *t* + 1; if so, the growth transition was derived from size measurements at both times. Fecundity was calculated as the ratio between the number of recruits at time *t* + 1 and the number of mature individuals at time *t* (Ellner and Rees 2006). This estimation method was chosen because *E. edulis* reproduces through fruit bunches, which hinders precise quantification of the actual number of flowers or fruits produced due to losses caused by disturbances (Matos and Watkinson 1998). Moreover, as a long‐lived species, fecundity has relatively low influence on demographic parameter estimation (Franco and Silvertown 2004).

Model selection was performed using the Akaike Information Criterion (AIC), relating survival and growth to individual size (i.e., size‐independent, size‐dependent, size^2^, and size^3^). Four models were tested: vital rate ~1, vital rate ~size, vital rate ~size + size^2^, and vital rate ~size + size^2^ + size^3^. The simplest model with the lowest AIC was selected when ΔAIC ≤ 2 (Tables S1 and S2). For Scenario 1, a total of 17 control kernels were generated for each population, each representing one transition year.

For Scenarios 2, 3, and 4, which included harvesting, kernels were also estimated by considering population structural variation during the sampling interval; however, additional mortality was simulated for individuals targeted under each harvesting scenario (i.e., mature individuals, mature and immature individuals, or fruits), at different harvesting intensities (30%, 60%, and 100%). These kernels are hereafter referred to as harvesting kernels. The harvesting simulation was implemented through the random selection of individuals within the targeted categories in each scenario, representing mortality. A bootstrap procedure with 2000 replicates was performed, where each loop simulated the death of a different combination of individuals. As a result, for each transition year of each population, 2000 harvesting kernels were generated.

Having estimated both the control kernels and the harvesting kernels for each transition year of each population, we calculated the stochastic population growth rate (*λ*_*S*_) using the function *stochGrowthRateSampleList* from the IPMpack package (Metcalf et al. 2013). This calculation involved 5000 iterations, in which, at each iteration, control and harvesting kernels were randomly sampled to total 10 kernels, representing a simulation of a 10‐year period. For the low‐frequency scenario, nine control kernels and one harvesting kernel were randomly selected, representing 9 years without harvesting and 1 year with harvesting. For the medium‐frequency scenario, seven control kernels and three harvesting kernels were randomly selected, while for the high‐frequency scenario, five control kernels and five harvesting kernels were randomly selected. At each iteration, one stochastic population growth rate was calculated, and at the end of the 5000 iterations, a mean *λ*_S_ value and its 95% confidence interval were estimated for each population. The *λ*_S_ value indicates the population trend over the analyzed period: if *λ*_S_ > 1, the population is projected to increase over the long term; if *λ*_S_ = 1, the population is projected to be stable over the long term; and if *λ*_S_ < 1, the population is projected to decline over the long term. However, if the 95% confidence interval for *λ*_S_ overlaps 1, the population is considered stable.

#### 2.5.2 Deterministic Dynamics

In the control scenario, deterministic population growth rates (*λ*_D_) were estimated in order to evaluate fluctuations in population values over the entire sampling period. To this end, the mean *λ*_D_ and its 95% confidence interval were calculated using a bootstrap procedure with 2000 replicates. In each iteration, 5% of the individuals from each transition year were randomly removed. At the end of each iteration, the removed individuals were returned to the population, and a new iteration was performed with a different random subset of individuals removed.

#### 2.5.3 Prospective Analysis

Elasticity analysis was used to evaluate which sizes and vital rates exerted the greatest influence on *λ* (de Kroon et al. 1986). Stochastic elasticity analyses were performed separately for each population, based on the time series represented by each control kernel. This approach decomposes elasticity into components associated with changes in the mean values of matrix elements and with temporal variability, providing a more detailed understanding of how vital rates influence *λ*_S_ under fluctuating environmental conditions (Haridas et al. 2009). All data analyses were conducted in R (R Development Core Team 2019) using the packages IPMpack (Metcalf et al. 2013), gamm4 (Wood et al. 2017), Rage (Jones et al. 2022), and fields (Nychka et al. 2015).

## 3 Results

**FIGURE 3:**
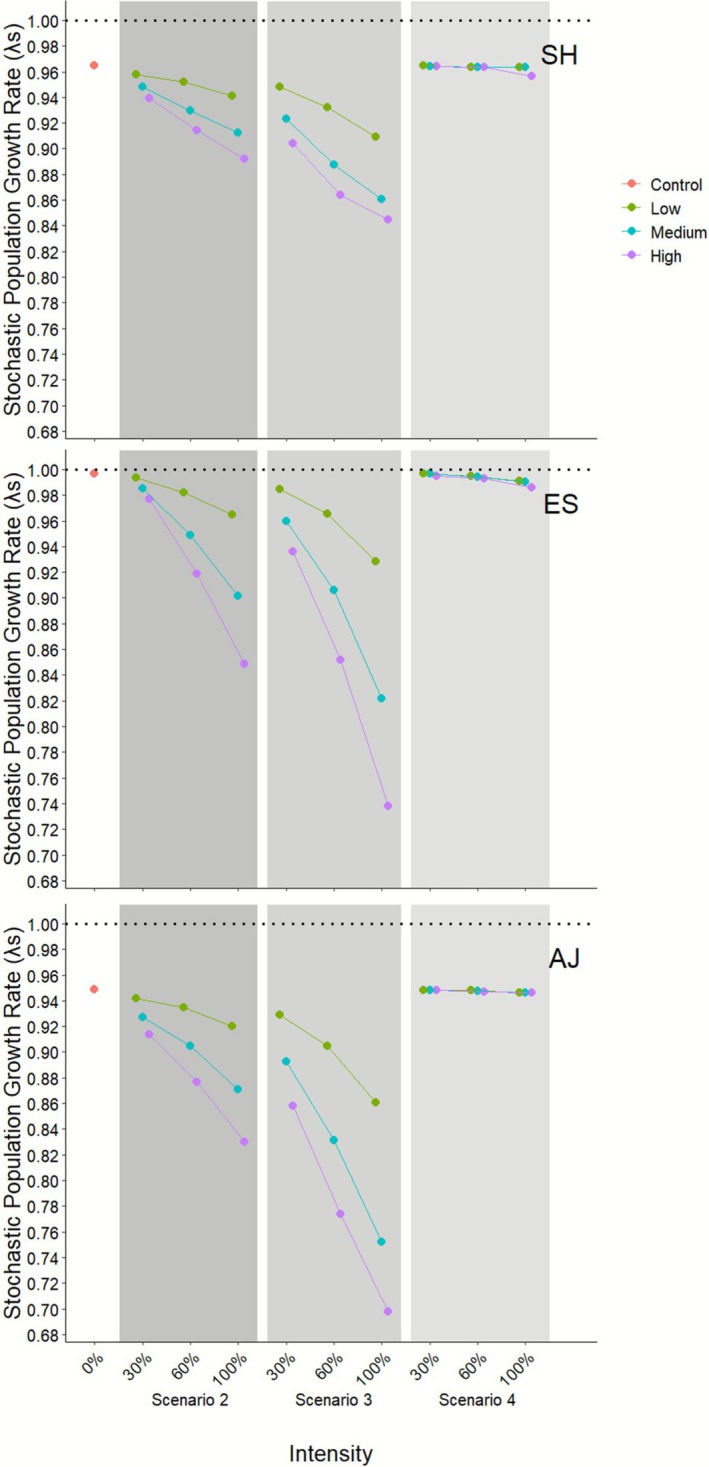
Stochastic population growth rates (*λ*_S_) for three populations of *Euterpe edulis* Santa Helena (SH), Estreito (ES), and Afetiva‐Jorge (AJ), sampled in three forest fragments in Silva Jardim, Rio de Janeiro, Brazil. The graph presents *λ*_S_ values for four scenarios: Control (scenario 1, white background), simulation of harvesting involving only mature individuals (scenario 2, dark gray background), harvesting involving both immature and mature individuals (scenario 3, gray background), and fruit harvesting (scenario 4, light gray background). For each scenario, three different removal intensities (30%, 60%, and 100%) were applied, under varying frequencies of harvesting events: Low (one event every 10 years), Medium (three events every 10 years), and High (five events every 10 years). The black horizontal dashed line at *λ*_S_ = 1.0 is used as a reference for neutral population growth.

**FIGURE 4:**
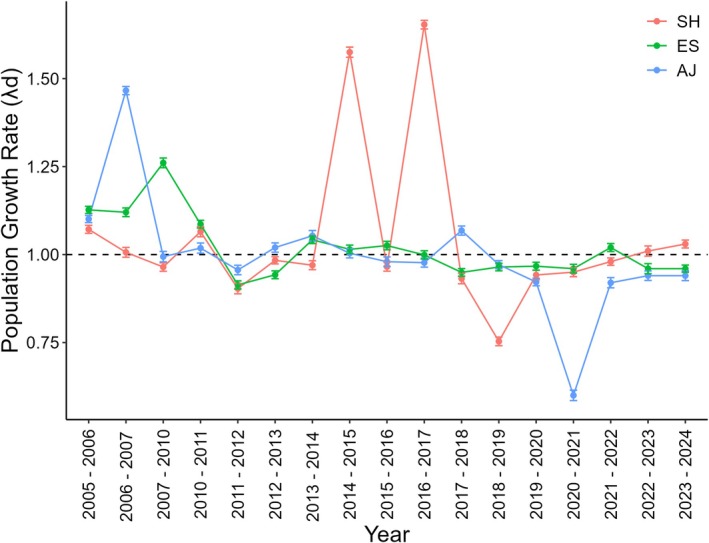
Deterministic population growth rates (λ_D_) for the Santa Helena (SH), Estreito (ES), and Afetiva‐Jorge (AJ) populations, sampled over a 19‐year period in forest fragments of the Brazilian Atlantic Forest. The 95% confidence intervals for each value are shown at each point.

In the control scenario, the three populations declined throughout the study period (Figure 3). The ES population showed the mildest decline, with *λ*_S‐ES_ = 0.9970 (±0.0010), representing a reduction of < 1% over the 19‐year sampling period. During this period, ES exhibited a maximum deterministic *λ*_D‐ES_ of 1.2606 (±0.0013) and a minimum of 0.9138 (±0.0011) (Figure 4). In contrast, the SH and AJ populations displayed more pronounced declining trends during the sampling period, with *λ*_S‐SH_ = 0.9647 (±0.0015) and *λ*_S‐AJ_ = 0.9489 (±0.0016), respectively. The SH population showed a maximum deterministic *λ*_D‐SH_ of 1.6533 (±0.0010) and a minimum of 0.7534 (±0.0014), while AJ exhibited a maximum λ_D‐AJ_ of 1.4660 (±0.0015) and a minimum of 0.6035 (±0.0010) (Figure 4).

Harvesting of palm heart targeting only mature individuals (scenario 2) showed a steadily increasing decline in *λ*_S_ as removal intensity increased. In all three populations, at a 30% removal intensity, *λ*_S_ decreased compared to the control, but there was little variation across different harvesting event frequencies (Figure 3). At a 60% removal intensity, the low‐frequency scenario exhibited less variation in *λ*_S_ than the 30% removal frequency, with a gradual increase in variation as the harvesting frequency and intensity increased. In the worst‐case harvesting scenario, with 100% removal intensity and high harvesting frequency, targeting only mature individuals, the lowest *λ*_S_ values were observed, with *λ*_S‐SH_ = 0.8917 (±0.0026), *λ*_S‐ES_ = 0.8484 (±0.0016), and *λ*_S‐AJ_ = 0.8297 (±0.0018).

In scenario 3, which involved harvesting individuals with DGL ≥ 65 mm, the trend was similar to that observed in scenario 2, with a decrease in *λ*_S_ as both the intensity and frequency of harvesting events increased (Figure 3). At a 30% removal intensity, the range of variation between observed values was smaller, increasing with the frequency of harvesting events. The *λ*_S_ values were highest under low‐frequency events and 30% intensity, with *λ*_S‐SH_ = 0.9482 (±0.0015), *λ*_S‐ES_ = 0.9847 (±0.0011), and *λ*_S‐AJ_ = 0.9291 (±0.0017). The lowest *λ*_S_ values were observed under high‐frequency events and 100% intensity, with *λ*_S‐SH_ = 0.8447 (±0.0026), *λ*_S‐ES_ = 0.7381 (±0.0018), and *λ*_S‐AJ_ = 0.6977 (±0.0027).

In scenario 4, there was little variation in the *λ*_S_ values, regardless of the intensity and frequency of harvesting events. The highest values were observed under low‐frequency harvesting events and 30% intensity, with *λ*_S‐SH_ = 0.9646 (±0.0015), *λ*_S‐ES_ = 0.9967 (±0.0020), and *λ*_S‐AJ_ = 0.9483 (±0.0017). The lowest values were observed under high‐frequency harvesting events and 100% intensity, with *λ*_S‐SH_ = 0.9563 (±0.0003), *λ*_S‐ES_ = 0.9858 (±0.0008), and *λ*_S‐AJ_ = 0.9462 (±0.0004).

**FIGURE 5:**
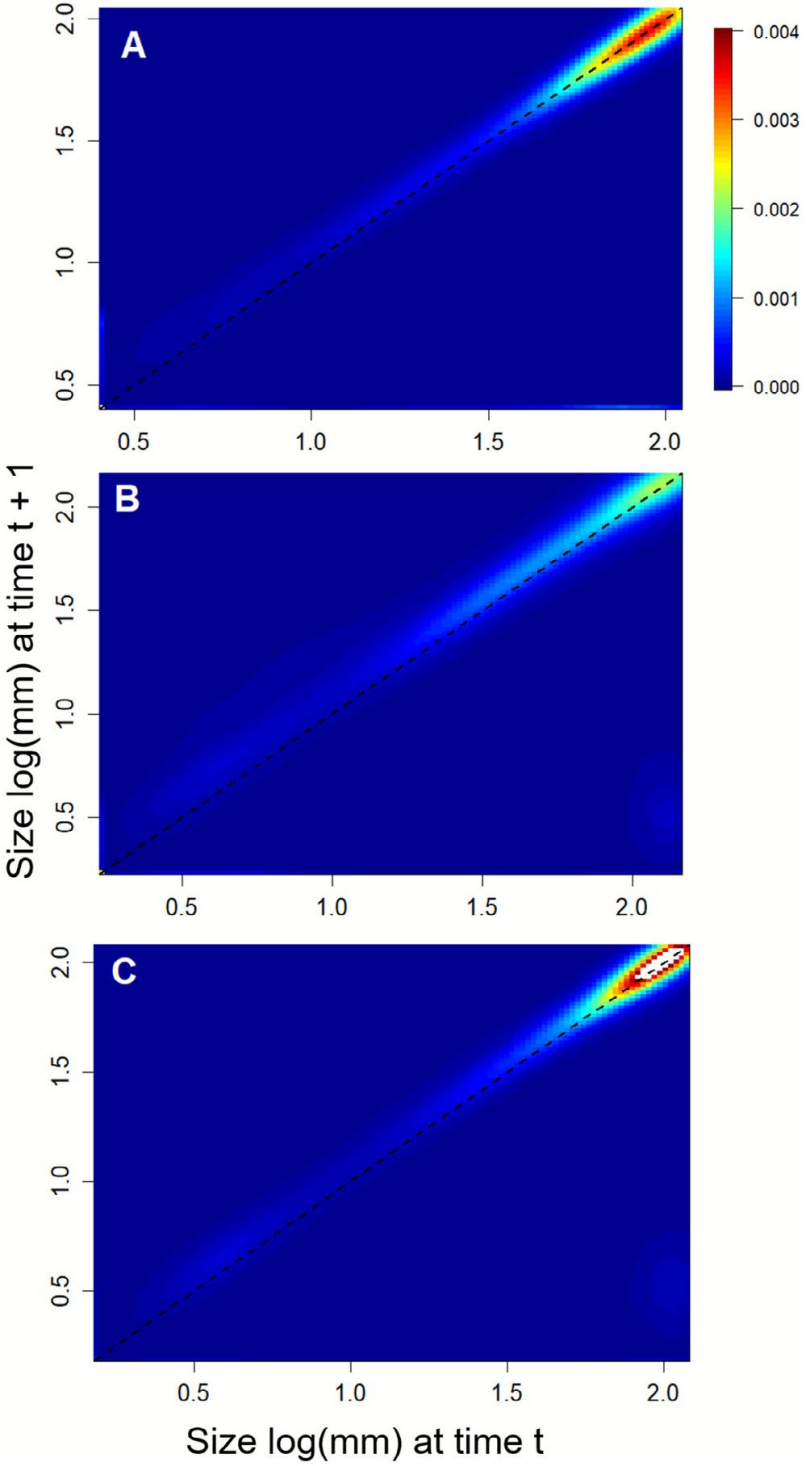
Stochastic elasticity for each of the three sampled populations, Santa Helena (A), Estreito (B), and Afetiva‐Jorge (C) The *X*‐axis represents the logarithmic scale of individual sizes in the population at time *t*, while the *Y*‐axis represents the logarithmic scale of individual sizes at time *t* + 1. Warmer colors indicate transitions of greater importance, whereas cooler colors indicate transitions of lesser importance. The diagonal dashed black line serves as a reference for the probability of stasis.

Overall, for each of the three sampled populations, the stasis of larger individuals (i.e., > 1.7 mm in log scale) was the vital rate with the greatest influence on *λ*_S_. The vital rates associated with smaller individuals (i.e., < 1.7 mm on a log scale), as well as fecundity, had low influence on *λ*_S_ (Figure 5A–C).

## 4 Discussion

The palm *Euterpe edulis* is susceptible to various anthropogenic pressures, like habitat destruction, fragmentation, habitat isolation, palm heart and fruit harvesting. Based on the results of this study, we observed that the populations are demographically declining over recent years. The population reduction trend was evident under the control scenario, possibly in response to land‐use alterations such as fragmentation and forest loss. When the effect of harvesting was incorporated into the demographic analysis, the decline trend worsened, especially at high harvesting intensities and frequencies.

Low individual density was observed across the three sampled populations. Even in the ES population, which exhibited the highest overall density among the three populations, the value remains significantly lower than in other species' populations in forested areas with minimal anthropogenic influence (e.g., continuous forests and protected areas; Souza et al. 2020). When assessing the density of mature individuals, it is evident that the populations do not reach even half of the threshold required to maintain a viable population. According to Freckleton et al. (2003), based on calculations of population density and harvesting rates of individuals, a viable population of *E. edulis* must have a minimum density of 60 mature individuals per hectare. Thus, the already low individual densities in the three sampled populations, combined with the ongoing anthropogenic impacts, underscore the importance of effective monitoring and well‐designed conservation strategies for populations within small and unprotected forest remnants.

The species *E. edulis* occurs only within preserved forests (Henderson et al. 1995). In the control scenario, although ES exhibited a higher *λ*_S_ and SH and AJ lower values, we observed an overall trend of population decline across the three populations during the sampling years. This shows that land‐use changes in the region where the populations are located likely had a decisive impact on the species' persistence in the forest fragments. In the same region where the data for this study were collected, previous studies had already shown a similar declining trend, also in fragmented forest areas (Portela et al. 2010a, 2010b).

Although under protection, the region where the sampled populations are located has been subjected for decades to intense land‐use changes, including habitat loss due to the expansion of urban areas and agricultural and livestock activities (Vancine et al. 2024). Because of ecosystem alterations, *E. edulis* populations in some areas of the region have been suffering from over‐predation by animals (e.g., *Cebus nigritus* —Capuchin monkeys), likely due to a lack of alternative food resources for these animals, further exacerbating the demographic impact (Portela and Dirzo 2020). In our three populations, we did not detect this interaction during the study; however, the populations remain vulnerable to this risk in the future. On the other hand, populations in areas with greater forest cover and lower susceptibility to anthropogenic pressure appear to be demographically stable, exhibiting high densities of individuals and exceeding the threshold of 60 mature individuals per hectare (Souza and Prevedello 2020, Leal et al. 2021; see Freckleton et al. 2003).

When simulating harvesting on the populations, the demographic trend of decline becomes even more severe. In scenarios of low intensity and low frequency of harvesting events, the population shows some tolerance, with no decline in population growth rate values, similar to what was observed in a previous study for the species (Mendes et al. 2022). However, at high intensities of harvesting and more frequent removal events, the demographic situation of the populations becomes drastic, potentially reducing them by up to 30%, as observed in the results. Based on the behavior of harvesters, it is understood that situations of high intensity and frequency of harvesting are the most likely to occur in practice (Galetti and Fernandez 1998; Jones et al. 2008; Raymundo and Caballes 2016). Therefore, it is crucial that enforcement actions against illegal harvesting are conducted in the species' areas of occurrence and that penalties are appropriately applied to violators. Currently, harvesting of *E. edulis* palm heart is considered a crime under the Atlantic Forest Law (Law No. 11428/2006), a law that for almost two decades has been providing guidelines on the conservation, preservation, and management of Atlantic Forest resources.

Harvesting involving the removal of larger individuals from the population, as seen in scenarios 2 and 3, led to a more pronounced change in *λ*_S_ values, confirming our first hypothesis. This outcome was expected, as in long‐lived species, the survival of larger individuals in a population has the greatest influence on population growth rates (Franco and Silvertown 2004). Palm heart harvesting, as a method that involves killing individuals, is a decisive factor in the abrupt decline in *λ*_S_ values (Ticktin 2004; Schmidt et al. 2011). As observed in the elasticity results for this study, the largest individuals in the three populations had the highest impact on *λ*_S_ values throughout the sampling period.

On the other hand, fruit harvesting was shown to be less demographically harmful to *E. edulis* populations, even at high intensities and frequencies of harvesting, confirming our second hypothesis. This type of harvesting, by allowing mature individuals to remain alive in the population, has a lower impact on *λ*_S_ (Ticktin 2004, Schmidt et al. 2011). Here, the vital rate affected by this type of harvesting is fecundity, commonly of lesser importance in long‐lived species (Franco and Silvertown 2004). As observed in the stochastic elasticity results across the three populations, fecundity remained low throughout the sampling period, suggesting that fruit harvesting is not a primary driver of population decline. Nonetheless, harvesting effects are not always strictly negative, as density‐dependent mechanisms, particularly acting at early life stages in *E. edulis* , may attenuate some impacts in low‐density populations. Other studies highlight the low demographic impact of non‐destructive harvesting on populations of various target species, such as leaf harvesting (Ticktin et al. 2002; Endress et al. 2006), seed harvesting (Zuidema and Boot 2002; Mendes et al. 2022), and bark harvesting (Guedje et al. 2007; Gaoue and Ticktin 2010).

Fruit harvesting has been shown to be less demographically harmful to population persistence. However, the distribution area of *E. edulis* is along a region of high anthropogenic pressure, with large cities and the establishment of extensive areas for livestock and agriculture (Souza and Prevedello 2019; Souza et al. 2020). Currently, only 23% of the native vegetation remains available for occupation by the species (Vancine et al. 2024). As a result, most *E. edulis* populations are composed of a few individuals, mainly those in the reproductive stage (Melito et al. 2014; Souza and Prevedello 2020). This situation suggests that fruit harvesting may be detrimental to the persistence of species populations, reducing regeneration and the recruitment of new individuals (Avocèvou‐Ayisso et al. 2009). Furthermore, it may negatively affect ecosystem functioning (Muler et al. 2014; Picchio et al. 2020; Santos et al. 2023). As *E. edulis* serves as a key resource for several fauna species, particularly during periods when other plant species are not fruiting, the lack of resource provision can have cascading impacts on wildlife. Since many of these animals act as seed dispersers of *E. edulis* , the disruption of seed dispersal among meta‐populations may lead to genetic consequences such as reduced gene flow, increased inbreeding, and bottleneck effects (Pereira et al. 2022; Santos et al. 2016). Additionally, the arrival of seeds from other species into areas where these populations occur may be compromised, potentially driving homogenization within the local plant community (Lôbo et al. 2011).

Despite these effects associated with *E. edulis* fruit harvesting, human consumption of this resource has been increasingly consolidated. Owing to its high nutritional value (Pereira et al. 2023; Schulz et al. 2016), its uses are already widely observed in Brazilian cuisine, as well as in the medicinal and cosmetic industries (Morais et al. 2022; Siqueira et al. 2024). Therefore, it is essential to adopt fruit management strategies that promote sustainable consumption, preventing drastic consequences for species populations and their associated cascading effects while also enabling income generation for human communities that depend on the commercialization of these resources.

The three populations sampled in this study are located within a protected area (APA‐SJ) and in a social context where enforcement against illegal harvesting and alteration of remaining forested areas is stringent. Nonetheless, some cases of palm heart harvesting were detected, and the expansion of urban areas and agricultural activities is also a contributing factor. Only 30% of the Brazilian Atlantic Forest remains under protected status, placing other remnant populations at even greater risk of extinction due to anthropogenic pressures, including forest destruction and illegal harvesting (Vancine et al. 2024). Furthermore, the Human Development Index (HDI) of the municipalities within the APA‐SJ (i.e., Silva Jardim, Rio Bonito, Casemiro de Abreu, and Rio das Ostras) is high relative to the Brazilian HDI (PNUD 2013). This is an additional positive factor, as it suggests that the resident human population in these municipalities has access to quality goods and services, reducing the need to engage in illegal forest resource harvesting for subsistence or income generation (Price and Gittleman 2007; Stanley et al. 2012).

Our harvest simulations assumed fixed vital‐rate functions following removals, without updating the post‐harvest crowding environment experienced by the surviving individuals. Consequently, potential density‐dependent mechanisms were not incorporated. In the context of this study, because populations are small, density‐dependent effects are unlikely to occur. However, in studies involving larger populations, this factor should be taken into account, as it may lead to demographic outcomes that deviate from the true population responses (Freckleton et al. 2003; Haugen et al. 2007). Additionally, all demographic data were collected after the fruiting and dispersal period (Portela et al. 2020), potentially omitting variation in recruitment and early survival that could be detected through pre‐fruiting censuses (Freckleton et al. 2003). Although fecundity has low elasticity in long‐lived species such as *E. edulis* , suggesting limited influence on *λ*, integrating both pre‐ and post‐fruiting monitoring could refine estimates of demographic transitions. These considerations highlight the need for caution when interpreting the management implications derived from our simulations.

## 5 Conclusion

The management and conservation of *Euterpe edulis* are complex, requiring environments with preserved forest characteristics, and they are also demographically susceptible to resource harvesting. Even in regions where enforcement of land‐use changes is intensive, populations showed demographic reductions in the control scenario. When palm heart harvesting is incorporated into the population dynamics, the demographic decline becomes even more severe, particularly when it occurs at high intensities and more frequently. On the other hand, fruit harvesting appears to be less demographically harmful to population persistence; however, community and ecosystem responses need to be further evaluated. Fruit collection was less demographically influenced, allowing it to occur under specific conditions and including incentives for new plantings aimed at responsible production.

In a broader context, the conservation of the Atlantic Forest, a severely reduced ecosystem, demands immediate management and conservation measures. With more than half of the remaining forest located outside protected areas, species that depend on forests for survival face many challenges in fragmented landscapes. Therefore, long‐term monitoring is crucial for developing effective conservation strategies.

Some recommendations and implications arising from this study are:

1. Harvesting of reproductive structures (e.g., fruits) can be demographically sustainable under carefully regulated conditions, but ecological interactions and community‐level impacts must be assessed before widespread implementation.
2. Cultivation of target species, such as *E. edulis* , should be promoted as an alternative to wild harvesting, especially in an agroforestry context and for non‐destructive uses such as fruit production.
3. Palm heart harvesting must remain strictly prohibited, particularly in small and fragmented forest patches, where our simulations show it leads to rapid and irreversible population collapse. Enforcement should be strengthened to prevent illegal exploitation in these high‐risk areas.
4. There is a pressing need for national‐level policies and legal frameworks focused on the sustainable management and conservation of non‐timber forest products, with particular attention to populations in fragmented and unprotected landscapes.

Ultimately, this study highlights how demographic modeling can guide sustainable resource use and conservation strategies across different species and ecosystems. In highly fragmented tropical forests, where many plant populations are already operating near viability thresholds, even minimal additional pressures can lead to extinction. Long‐term demographic monitoring is essential for anticipating such tipping points and ensuring the persistence of forest‐dependent species.

## Author Contributions

**Eduardo T. B. Mendes:** conceptualization (equal), data curation (equal), formal analysis (equal), investigation (equal), methodology (equal), project administration (equal), validation (equal), writing – original draft (equal), writing – review and editing (equal). **Rita C. Q. Portela:** conceptualization (equal), formal analysis (equal), funding acquisition (equal), methodology (equal), project administration (equal), resources (equal), supervision (equal), validation (equal), visualization (equal), writing – review and editing (equal).

## Conflicts of Interest

The authors declare no conflicts of interest.

## Supporting information


**Table S1:** Models describing the effect of plant size on survival for each year interval at each studied population of *Euterpe edulis*: SH, AJ and ES, Rio de Janeiro, Brazil. ∆AIC: difference between the Akaike Information Criterion (AIC) of a given model and the best model. ∆AIC ≤ 2 indicates equally plausible models. When more than a model was considered plausible, only the simplest one (highlighted in bold) was considered for the population dynamic description through the Integral Projection Model (IPM).
**Table S2:** Models describing the effect of plant size on growth for each year interval at each studied population of *Euterpe edulis*: SH, AJ and ES, Rio de Janeiro, Brazil. ∆AIC: difference between the Akaike Information Criterion (AIC) of a given model and the best model. ∆AIC ≤ 2 indicates equally plausible models. When more than a model was considered plausible, only the simplest one (highlighted in bold) was considered for the population dynamic description through the Integral Projection Model (IPM).

## Data Availability

All data used in this study, including the codes used in R, are available at Figshare: https://figshare.com/s/3e7c791cfd82bb0f9a06.
