# Supplementary material for: Balancing Harvesting and Conservation: Demographic Responses of a Threatened Palm to Anthropogenic Disturbance
Source: Ecol Evol. 2026 Jan 7;16(1):e72882. doi: 10.1002/ece3.72882 (PMC12779404; doi:10.1002/ece3.72882)
Supplement: Supplementary file 1 — Table S1: Models describing the effect of plant size on survival for each year interval at each studied population of Euterpe edulis: SH, AJ and ES, Rio de Janeiro, Brazil. ∆AIC: difference between the Akaike Information Criterion (AIC) of a given model and the best model. ∆AIC ≤ 2 indicates equally plausible models. When more than a model was considered plausible, only the simplest one (highlighted in bold) was considered for the population dynamic description through the Integral Projection Model (IPM). Table S2: Models describing the effect of plant size on growth for each year interval at each studied population of Euterpe edulis: SH, AJ and ES, Rio de Janeiro, Brazil. ∆AIC: difference between the Akaike Information Criterion (AIC) of a given model and the best model. ∆AIC ≤ 2 indicates equally plausible models. When more than a model was considered plausible, only the simplest one (highlighted in bold) was considered for the population dynamic description through the Integral Projection Model (IPM). [file ECE3-16-e72882-s001.docx]

**Supplementary Material**

**Table S1.** Models describing the effect of plant size on survival for each year interval at each studied population of *Euterpe edulis*: SH, AJ and ES, Rio de Janeiro, Brazil. ∆AIC: difference between the Akaike Information Criterion (AIC) of a given model and the best model. ∆AIC ≤ 2 indicates equally plausible models. When more than a model was considered plausible, only the simplest one (highlighted in bold) was considered for the population dynamic description through the Integral Projection Model (IPM).

| **Survival** | | | | | | |
| --- | --- | --- | --- | --- | --- | --- |
| **Year interval** | **Santa Helena** | | **Afetiva-Jorge** | | **Estreito** | |
|  | **Model** | **ΔAIC** | **Model** | **ΔAIC** | **Model** | **ΔAIC** |
| 2005 – 2006 | **surv ~ 2.05size** | 0.0 | surv ~ size + size² | 0.0 | surv ~ size | 0.0 |
|  | surv ~ size + size² | **1.6** | **surv ~ 7.68size** | **0.2** | surv ~ size + size² | 0.5 |
|  | surv ~ size + size²+ size³ | 3.2 | surv ~ size + size²+ size³ | 1.8 | **surv ~ 1** | **1.5** |
|  | surv ~ 1 | 9.5 | surv ~ 1 | 29.4 | surv ~ size + size²+ size³ | 2.4 |
|  |  |  |  |  |  |  |
| 2006 – 2007 | **surv ~ 1.79size** | **0.0** | **surv ~ 1** | **0.0** | **surv ~ 1** | **0.0** |
|  | surv ~ size + size² | 0.5 | surv ~ size | 1.1 | surv ~ size + size² | 0.1 |
|  | surv ~ size + size²+ size³ | 1.3 | surv ~ size + size² | 2.1 | surv ~ size | 1.1 |
|  | surv ~ 1 | 3.3 | surv ~ size + size²+ size³ | 3.5 | surv ~ size + size²+ size³ | 1.9 |
|  |  |  |  |  |  |  |
| 2007 – 2010 | **surv ~ 2.97size** | **0.0** | **surv ~ 8.98size -3.10 size²** | **0.0** | surv ~ size + size²+ size³ | 0.0 |
|  | surv ~ size + size²+ size³ | 1.3 | surv ~ size + size²+ size³ | 2.0 | **surv ~ 8.12size -2.71size²** | **1.4** |
|  | surv ~ size + size² | 1.7 | surv ~ size | 3.8 | surv ~ size | 7.5 |
|  | surv ~ 1 | 14.8 | surv ~ 1 | 13 | surv ~ 1 | 25.2 |
|  |  |  |  |  |  |  |
| 2010 – 2011 | **surv ~ 1** | **0.0** | **surv ~ 4.31size** | **0.0** | **surv ~ -3.13size + 3.21size²** | **0.0** |
|  | surv ~ size | 0.2 | surv ~ size + size² | 1.7 | surv ~ size + size²+ size³ | 1.0 |
|  | surv ~ size + size² | 1.0 | surv ~ size + size²+ size³ | 3.7 | surv ~ size | 6.0 |
|  | surv ~ size + size²+ size³ | 3.0 | surv ~ 1 | 69.8 | surv ~ 1 | 35.9 |
|  |  |  |  |  |  |  |
| 2011 – 2012 | **surv ~ 3.64size** | **0.0** | surv ~ size + size² | 0.0 | **surv ~ -10.48size + 7.05size²** | **0.0** |
|  | surv ~ size + size² | 2.0 | **surv ~ 4.17size** | **0.3** | surv ~ size + size²+ size³ | 0.1 |
|  | surv ~ size + size²+ size³ | 4.0 | surv ~ size + size²+ size³ | 1.7 | surv ~ size | 13.6 |
|  | surv ~ 1 | 43.8 | surv ~ 1 | 58.7 | surv ~ 1 | 50.8 |
|  |  |  |  |  |  |  |
| 2012 – 2013 | surv ~ size + size²- size³ | 0.0 | **surv ~ 2.89size** | **0.0** | **surv ~ 1.50size** | **0.0** |
|  | **surv ~ 1** | **0.4** | surv ~ size + size² | 1.7 | surv ~ size + size² | 1.5 |
|  | surv ~ size | 2.2 | surv ~ size + size²+ size³ | 2.2 | surv ~ size + size²+ size³ | 3.1 |
|  | surv ~ size + size² | 3.5 | surv ~ 1 | 10.5 | surv ~ 1 | 8.2 |
|  |  |  |  |  |  |  |
| 2013 – 2014 | **surv ~1.34size** | **0.0** | **surv ~ 2.91size** | **0.0** | **surv ~ 3.79size** | **0.0** |
|  | surv ~ size + size² | 1.9 | surv ~ size + size² | 0.6 | surv ~ size + size² | 1.9 |
|  | surv ~ 1 | 2.6 | surv ~ size + size²+ size³ | 2.2 | surv ~ size + size²+ size³ | 3.9 |
|  | surv ~ size + size²+ size³ | 3.1 | surv ~ 1 | 3.7 | surv ~ 1 | 25.6 |
|  |  |  |  |  |  |  |
| 2014 – 2015 | **surv ~ 4.52size** | **0.0** | **surv ~ 6.80size** | **0.0** | **surv ~ 3.97size** | **0.0** |
|  | surv ~ size + size² | 1.3 | surv ~ size + size² | 2.0 | surv ~ size + size² | 0.2 |
|  | surv ~ size + size²+ size³ | 2.1 | surv ~ size + size²+ size³ | 3.2 | surv ~ size + size²+ size³ | 2.1 |
|  | surv ~ 1 | 36.7 | surv ~ 1 | 47.1 | surv ~ 1 | 31.2 |
|  |  |  |  |  |  |  |
| 2015 – 2016 | **surv ~ 1.70size** | **0.0** | **surv ~ 12.89size -4.40size²** | **0.0** | surv ~ size + size²+ size³ | 0.0 |
|  | surv ~ size + size² | 1.9 | surv ~ size + size²+ size³ | 1.6 | **surv ~ 2.43size** | **0.3** |
|  | surv ~ 1 | 3.4 | surv ~ size | 4.4 | surv ~ size + size² | 0.9 |
|  | surv ~ size + size²+ size³ | 3.5 | surv ~ 1 | 21.5 | surv ~ 1 | 15.0 |
|  |  |  |  |  |  |  |
| 2016 – 2017 | **surv ~ 1.81size** | **0.0** | **surv ~ 2.47size** | **0.0** | **surv ~ 115.38size -165.57size²+ 75.65size³** | **0.0** |
|  | surv ~ size + size² | 1.1 | surv ~ size + size²+ size³ | 0.5 | surv ~ size + size² | 2.5 |
|  | surv ~ size + size²+ size³ | 2.3 | surv ~ size + size² | 1.9 | surv ~ size | 10.8 |
|  | surv ~ 1 | 3.6 | surv ~ 1 | 14.9 | surv ~ 1 | 61.0 |
|  |  |  |  |  |  |  |
| 2017 – 2018 | **surv ~ 7.54size -2.36 size²** | **0.0** | surv ~ size + size² | **0.0** | **surv ~ 8.95size -2.90size²** | **0.0** |
|  | surv ~ size + size²+ size³ | 0.7 | **surv ~ 2.58 size** | 0.1 | surv ~ size + size²+ size³ | 1.8 |
|  | surv ~ size | 4.4 | surv ~ size + size²+ size³ | 2.0 | surv ~ size | 3.4 |
|  | surv ~ 1 | 88.7 | surv ~ 1 | 16.4 | surv ~ 1 | 11.5 |
|  |  |  |  |  |  |  |
| 2018 – 2019 | **surv ~ 72.77size -48.12 size²+ 0.53size³** | **0.0** | **surv ~ 31.53size -26.03size²+ 6.65size³** | **0.0** | **surv ~ 3.17size** | **0.0** |
|  | surv ~ size + size² | 2.2 | surv ~ size + size² | 2.7 | surv ~ size + size² | 1.2 |
|  | surv ~ size | 4.7 | surv ~ size | 7.3 | surv ~ size + size²+ size³ | 2.7 |
|  | surv ~ 1 | 57.7 | surv ~ 1 | 32.2 | surv ~ 1 | 48.2 |
|  |  |  |  |  |  |  |
| 2019 – 2020 | **surv ~ 3.23size** | **0.0** | **surv ~ 2.30size** | **0.0** | surv ~ size + size²+ size³ | 0.0 |
|  | surv ~ size + size² | 0.6 | surv ~ size + size² | 0.9 | surv ~ size + size² | 1.6 |
|  | surv ~ size + size²+ size³ | 2.3 | surv ~ size + size²+ size³ | 2.9 | **surv ~ 1.71size** | **1.8** |
|  | surv ~ 1 | 51.3 | surv ~ 1 | 44.1 | surv ~ 1 | 14.0 |
|  |  |  |  |  |  |  |
| 2020 – 2021 | **surv ~ 16.05size - 5.14size²** | **0.0** | **surv ~ 1** | **0.0** | **surv ~ 2.20size** | **0.0** |
|  | surv ~ size + size²+ size³ | 2.0 | surv ~ size + size²+ size³ | 0.7 | surv ~ size + size² | 2.0 |
|  | surv ~ size | 4,2 | surv ~ size + size² | 1.4 | surv ~ size + size²+ size³ | 3.4 |
|  | surv ~ 1 | 14.2 | surv ~ size | 1.9 | surv ~ 1 | 25.9 |
|  |  |  |  |  |  |  |
| 2021 – 2022 | **surv ~ -5.73size + 3.30size²** | **0.0** | surv ~ size + size²+ size³ | 0.0 | surv ~ size + size²+ size³ | 0.0 |
|  | surv ~ size + size²+ size³ | 0.2 | **surv ~ 1.25size** | **0.2** | **surv ~ 3.94size – 4.95size²** | **0.9** |
|  | surv ~ size | 3.2 | surv ~ size + size² | 1.8 | surv ~ size | 5.6 |
|  | surv ~ 1 | 17.6 | surv ~ 1 | 6.5 | surv ~ 1 | 39.5 |
|  |  |  |  |  |  |  |
| 2022 – 2023 | **sizeNext ~ 1.09size** | **0.0** | **sizeNext ~ 2.16size** | **0.0** | **surv ~ -3.39size + 2.62size²** | **0.0** |
|  | sizeNext ~ size + size² | 0.7 | sizeNext ~ size + size²+ size³ | 0.3 | sizeNext ~ size + size²+ size³ | 0.4 |
|  | sizeNext ~ size + size²+ size³ | 1.2 | sizeNext ~ size + size² | 1.1 | surv ~ size | 3.9 |
|  | sizeNext ~ 1 | 2.9 | sizeNext ~ 1 | 29.6 | sizeNext ~ 1 | 42.3 |
|  |  |  |  |  |  |  |
| 2023 – 2024 | **surv ~ 1.83size** | **0.0** | **surv ~ 9.34size - 2.91size²** | **0.0** | **surv ~ 2.37size** | **0.0** |
|  | surv ~ size + size²+ size³ | **1.2** | surv ~ size + size²+ size³ | 1.9 | sizeNext ~ size + size² | 2.0 |
|  | surv ~ size + size² | 1.2 | surv ~ size | 5.3 | sizeNext ~ size + size²+ size³ | 3.6 |
|  | surv ~ 1 | 6.9 | surv ~ 1 | 50.8 | sizeNext ~ 1 | 24.8 |

**Table S2.** Models describing the effect of plant size on growth for each year interval at each studied population of *Euterpe edulis*: SH, AJ and ES, Rio de Janeiro, Brazil. ∆AIC: difference between the Akaike Information Criterion (AIC) of a given model and the best model. ∆AIC ≤ 2 indicates equally plausible models. When more than a model was considered plausible, only the simplest one (highlighted in bold) was considered for the population dynamic description through the Integral Projection Model (IPM).

| **Growth** | | | | | | |
| --- | --- | --- | --- | --- | --- | --- |
| Year interval | **Santa Helena** |  | **Afetiva-Jorge** |  | **Estreito** |  |
|  | **Model** | **ΔAIC** | **Model** | **ΔAIC** | **Model** | **ΔAIC** |
| 2005 – 2006 | **sizeNext ~ 0.94size** | **0.0** | **sizeNext ~ 0.89size** | **0.0** | **sizeNext ~ -0.18size + 0.99size²-0.26size³** | **0.0** |
|  | sizeNext ~ size + size² | 2.0 | sizeNext ~ size + size² | 1.2 | sizeNext ~ size | 2.3 |
|  | sizeNext ~ size + size²+ size³ | 2.3 | sizeNext ~ size + size²+ size³ | 1.4 | sizeNext ~ size + size² | 4.2 |
|  | sizeNext ~ 1 | 281.1 | sizeNext ~ 1 | 175.6 | sizeNext ~ 1 | 18.5 |
|  |  |  |  |  |  |  |
| 2006 – 2007 | **sizeNext ~ 2.17size -0.95size²+0.23size³** | **0.0** | **sizeNext ~ 0.96size** | **0.0** | sizeNext ~ size + size² | 0.0 |
|  | sizeNext ~ size | 9.8 | sizeNext ~ size + size² | 1.6 | sizeNext ~ size + size²+ size³ | 1.6 |
|  | sizeNext ~ size + size² | 10.6 | sizeNext ~ size + size²+ size³ | 3.5 | **sizeNext ~ size** | **1.7** |
|  | sizeNext ~ 1 | 300.9 | sizeNext ~ 1 | 198.4 | sizeNext ~ 1 | 37.9 |
|  |  |  |  |  |  |  |
| 2007 – 2010 | **sizeNext ~ 0.93size** | **0.0** | **sizeNext ~ 0.73size** | **0.0** | **sizeNext ~0.80 size** | **0.0** |
|  | sizeNext ~ size + size² | 1.8 | sizeNext ~ size + size²+ size³ | 0.0 | sizeNext ~ size + size² | 2.0 |
|  | sizeNext ~ size + size²+ size³ | 2.4 | sizeNext ~ size + size² | 0.6 | sizeNext ~ size + size²+ size³ | 3.4 |
|  | sizeNext ~ 1 | 144.2 | sizeNext ~ 1 | 78.8 | sizeNext ~ 1 | 17.8 |
|  |  |  |  |  |  |  |
| 2010 – 2011 | **sizeNext ~ size** | **0.0** | **sizeNext ~ 0.05size + 0.88size²-0.25 size³** | **0.0** | **sizeNext ~ 0.88size** | **0.0** |
|  | sizeNext ~ size + size² | 1.1 | sizeNext ~ size + size² | 12.1 | sizeNext ~ size + size² | 1.7 |
|  | sizeNext ~ 1.32size -0.30size²+ 0.07size³ | 1.6 | sizeNext ~ size | 15.5 | sizeNext ~ size + size²+ size³ | 2.0 |
|  | sizeNext ~ 1 | 234.7 | sizeNext ~ 1 | 141.6 | sizeNext ~ 1 | 119.8 |
|  |  |  |  |  |  |  |
| 2011 – 2012 | sizeNext ~ size + size²+ size³ | 0.0 | sizeNext ~ size + size²+ size³ | 0.0 | **sizeNext ~ 0.95size** | **0.0** |
|  | **sizeNext ~ 0.96size** | **0.6** | **sizeNext ~ 0.99size** | **0.2** | sizeNext ~ size + size²+ size³ | 1.6 |
|  | sizeNext ~ size + size² | 0.6 | sizeNext ~ size + size² | 2.2 | sizeNext ~ size + size² | 1.8 |
|  | sizeNext ~ 1 | 355.4 | sizeNext ~ 1 | 183.1 | sizeNext ~ 1 | 47.8 |
|  |  |  |  |  |  |  |
| 2012 – 2013 | **sizeNext ~ 0.95size** | **0.0** | sizeNext ~ size + size² | 0.0 | **sizeNext ~ 0.96size** | **0.0** |
|  | sizeNext ~ size + size² | 1.3 | sizeNext ~ size + size²+ size³ | 1.7 | sizeNext ~ size + size² | 1.1 |
|  | sizeNext ~ size + size²+ size³ | 2.6 | **sizeNext ~ 0.93size** | **1.9** | sizeNext ~ size + size²+ size³ | 2.9 |
|  | sizeNext ~ 1 | 274.2 | sizeNext ~ 1 | 165.8 | sizeNext ~ 1 | 36.7 |
|  |  |  |  |  |  |  |
| 2013 – 2014 | sizeNext ~ size + size²+ size³ | 0.0 | sizeNext ~ size + size² | 0.0 | **sizeNext ~ 0.72size + 0.07size²** | **0.0** |
|  | **sizeNext ~ 0.67size + 0.10size²** | **0.6** | **sizeNext ~ 0.95size** | **0.7** | sizeNext ~ size + size²+ size³ | 1.6 |
|  | sizeNext ~ size | 21.8 | sizeNext ~ size + size²+ size³ | 1.2 | sizeNext ~ size | 3.1 |
|  | sizeNext ~ 1 | 270.5 | sizeNext ~ 1 | 156.9 | sizeNext ~ 1 | 51.8 |
|  |  |  |  |  |  |  |
| 2014 – 2015 | sizeNext ~ size + size²+ size³ | 0.0 | **sizeNext ~0.92 size** | **0.0** | **sizeNext ~ 0.95size** | **0.0** |
|  | **sizeNext ~ 1.34size -0.12 size²** | **0.4** | sizeNext ~ size + size² | 1.4 | sizeNext ~ size + size²+ size³ | 1.5 |
|  | sizeNext ~ size | 5.2 | sizeNext ~ size + size²+ size³ | 2.0 | sizeNext ~ size + size² | 1.8 |
|  | sizeNext ~ 1 | 312.4 | sizeNext ~ 1 | 164.4 | sizeNext ~ 1 | 39.5 |
|  |  |  |  |  |  |  |
| 2015 – 2016 | **sizeNext ~ 0.68size + 0.09size²** | **0.0** | **sizeNext ~ 0.62size + 0.10size²** | **0.0** | **sizeNext ~ 0.26size + 0.53size²-0.13 size³** | **0.0** |
|  | sizeNext ~ size + size²+ size³ | 1.8 | sizeNext ~ size + size²+ size³ | 1.5 | sizeNext ~ size + size² | 2.2 |
|  | sizeNext ~ size | 4.1 | sizeNext ~ size | 4.1 | sizeNext ~ size | 4.0 |
|  | sizeNext ~ 1 | 189.9 | sizeNext ~ 1 | 75.4 | sizeNext ~ 1 | 65.4 |
|  |  |  |  |  |  |  |
| 2016 – 2017 | **sizeNext ~ 0.94size** | **0.0** | **sizeNext ~ -0.44size +1.06size²-0.25size³** | **0.0** | **sizeNext ~ 0.97size** | **0.0** |
|  | sizeNext ~ size + size² | 2.0 | sizeNext ~ size + size² | 9.1 | sizeNext ~ size + size²+ size³ | 2.6 |
|  | sizeNext ~ size + size²+ size³ | 3.9 | sizeNext ~ size | 10.0 | sizeNext ~ size + size² | 6.2 |
|  | sizeNext ~ 1 | 230.8 | sizeNext ~ 1 | 98.4 | sizeNext ~ 1 | 110.4 |
|  |  |  |  |  |  |  |
| 2017 – 2018 | **sizeNext ~ 0.20size+0.55size²-0.12 size³** | **0.0** | **sizeNext ~ -0.29size+0.78size²-0.16size³** | **0.0** | **sizeNext ~ 1.21size -0.08size²** | **0.0** |
|  | sizeNext ~ size + size² | 2.3 | sizeNext ~ size + size² | 3.9 | sizeNext ~ size + size²+ size³ | 0.3 |
|  | sizeNext ~ size | 10.1 | sizeNext ~ size | 12.4 | sizeNext ~ size | 8.6 |
|  | sizeNext ~ 1 | 64.0 | sizeNext ~ 1 | 172.4 | sizeNext ~ 1 | 77.3 |
|  |  |  |  |  |  |  |
| 2018 – 2019 | **sizeNext ~ 0.92size** | **0.0** | **sizeNext ~ 0.96size** | **0.0** | **sizeNext ~ 1.61size + 2.06size²-0.50size³** | **0.0** |
|  | sizeNext ~ size + size²+ size³ | 0.9 | sizeNext ~ size + size² | 1.9 | sizeNext ~ size | 7.8 |
|  | sizeNext ~ size + size² | 1.9 | sizeNext ~ size + size²+ size³ | 2.1 | sizeNext ~ size + size² | 8.4 |
|  | sizeNext ~ 1 | 221.2 | sizeNext ~ 1 | 98.4 | sizeNext ~ 1 | 126.9 |
|  |  |  |  |  |  |  |
| 2019 – 2020 | **sizeNext ~ 0.94size** | **0.0** | **sizeNext ~ size + size²** | **0.0** | **sizeNext ~ size + size²+ size³** | **0.0** |
|  | sizeNext ~ size + size²+ size³ | 0.8 | sizeNext ~ size + size²+ size³ | 2.0 | sizeNext ~ size | 4.7 |
|  | sizeNext ~ size + size² | 2.0 | sizeNext ~ size | 4.3 | sizeNext ~ size + size² | 5.4 |
|  | sizeNext ~ 1 | 145.2 | sizeNext ~ 1 | 63.5 | sizeNext ~ 1 | 13.7 |
|  |  |  |  |  |  |  |
| 2020 – 2021 | **sizeNext ~ 0.93size** | **0.0** | **sizeNext ~ -0.18size + 0.99size²- 0.25size³** | **0.0** | **sizeNext ~ -0.45size + 1.14size²- 0.27size³** | 0.0 |
|  | sizeNext ~ size + size²+ size³ | 2.0 | sizeNext ~ size + size² | 9.1 | sizeNext ~ size + size² | 18.0 |
|  | sizeNext ~ size + size² | 0.9 | sizeNext ~ size | 15,6 | sizeNext ~ size | 21.3 |
|  | sizeNext ~ 1 | 214.3 | sizeNext ~ 1 | 256.9 | sizeNext ~ 1 | 420.0 |
|  |  |  |  |  |  |  |
| 2021 – 2022 | **surv ~ 0.65size + 0.09size²** | **0.0** | **surv ~ 0.20size + 0.65size² - 0.17size³** | **0.0** | sizeNext ~ size + size²+ size³ | 0.0 |
|  | surv ~ size + size²+ size³ | 0.2 | surv ~ size + size² | 3.1 | **surv ~ 0.94size** | **0.3** |
|  | surv ~ size | 2.6 | surv ~ size | 3.7 | surv ~ size + size² | 0.9 |
|  | surv ~ 1 | 102.3 | surv ~ 1 | 270.0 | surv ~ 1 | 319.1 |
|  |  |  |  |  |  |  |
| 2022 – 2023 | **sizeNext ~ 0.91size** | **0.0** | **surv ~ 0.97size** | **0.0** | **sizeNext ~ 0.93size** | **0.0** |
|  | sizeNext ~ size + size² | 0.6 | surv ~ size + size² | 0.6 | sizeNext ~ size + size² | 2.0 |
|  | sizeNext ~ size + size²+ size³ | 2.3 | sizeNext ~ size + size²+ size³ | 2.5 | sizeNext ~ size + size²+ size³ | 2.4 |
|  | sizeNext ~ 1 | 90.2 | sizeNext ~ 1 | 253.3 | sizeNext ~ 1 | 50.2 |
|  |  |  |  |  |  |  |
| 2023 – 2024 | **sizeNext ~ 2.92size – 1.39size²+ 0.31size³** | **0.0** | sizeNext ~ size + size²+ size³ | 0.0 | sizeNext ~ size + size²+ size³ | 0.0 |
|  | sizeNext ~ size + size² | 5.2 | **sizeNext ~ 0.95size** | **1.1** | **sizeNext ~ 0.66size + 0.11size²** | **0.9** |
|  | sizeNext ~ size | 6.7 | sizeNext ~ size + size² | 2.5 | surv ~ size | 6.2 |
|  | sizeNext ~ 1 | 121.5 | sizeNext ~ 1 | 301.7 | surv ~ 1 | 326.3 |
